# Toxæmia and Insanitv

**Published:** 1901-01-05

**Authors:** 


### The Hospital

A Journal of

The Medical Sciences and Hospital Administration.

Vol. XXIX.—No. 745.] Edited by Sir Henry Burdett, K.C.B., and Solomon C. Smith, M.D., M.R.C.P. [January 5, 1901.

### Toxæmia and Insanity.

A highly instructive address on "Mental Disorders Dependent on Toxæmia," delivered by Sir Dyce Duckworth before the Medico-Psychological Association of Great Britain and Ireland, has just been published, and is well calculated to stimulate thought with regard to the grave questions with which it deals. Perhaps the most important event of the nineteenth century—certainly the most important in its scientific history, and probably also in relation to the future welfare of mankind—has been the revolution in pathology produced by the labours and discoveries of Pasteur. The intrusion of a chemist into their domain was strongly resented, and his conclusions were strenuously opposed by some of the intellectual pygmies who held high places in the worlds of medicine and surgery, but the march of the giant was irresistible, and the forces of truth were overwhelming. It has become a commonplace of daily knowledge that many forms of disease are produced by the intrusion of microbes into the body, or by the action of poisons which are formed in the course of their vital operations. It is probable that to the list of these diseases various forms of insanity will before long be added, or that, if they be not traced to poisons produced by microbes, they will be traced to poisons arising from irregular or imperfect performance of the changes incidental to the removal of waste and the repair of tissue. Ten years have passed away since an endeavour to establish this doctrine and to carry it to its legitimate consequences was made by a small section of the first London County Council. A Committee was appointed to inquire into the advantages which might be expected from the establishment of a hospital with a visiting medical staff for the study and curative treatment of insanity; and the Committee unanimously reported in favour of the proposal. The report pointed out, very much as Sir Dyce Duckworth has done in the address to which we refer, that phenomena resembling those of insanity are produced by the artificial production of toxic states of the blood, by alcohol, by opium, by chloroform, and many other agents, and that for a time these phenomena disappear when the toxic agents which produced them are eliminated from the system. On the other hand, if the toxic agents are continuously administered, structural alterations occur in the nervous system, and recovery becomes unlikely or impossible. It was suggested that the production and the ultimate incurability of insanity might be traced to a similar or analogous course of events. In order to determine whether this was so, resources beyond those of an "asylum" were required. The first necessity was a completely equipped clinical laboratory, under the control of a man of adequate skill and knowledge, who should search for evidence of the changes in the tissues and secretions which might be expected to occur; the second was the observation of the patients and of their symptoms in early stages of the disease, with constant reference to the facts ascertained in the laboratory, by a clinical physician of trained faculties and carefully garnered experience. The third necessity was a pathologist, whose chief business would be the investigation of post-mortem changes. None of these necessaries could be obtained in asylums, because the medical officers attached to them were too much employed in the business of administering large establishments to be able to devote their lives to laboratory or clinical work, and were moreover selected by magistrates and other unskilled people, with more reference to their past records as administrators than to any other consideration. The County Council Committee, therefore, strongly recommended the establishment of the proposed hospital. The Council, as a body, however, had no inducement to seize the opportunity. At the instance of the Asylums Committee, a gentleman of high attainments was appointed to be pathologist to the existing asylums of the Council, and this is all that so far has been done. It is a step in the right direction, but only a small one. The pathologist will throw light upon the structural changes incidental to advanced insanity, and will be a sort of scientific detective, telling us, after a burglary has been committed, the direction in which the thieves have made off with their booty. We want the clinical physician, and the clinical laboratory, to tell us when the thieves are prowling around with intention to break in. Above all, we want to get rid of the word "psychology," and of those who are misled by it. It is an *ignis fatuus.* We know of thought as a function of the brain, and as a function constantly disturbed by changes in its structure or in its blood supply, and these are proper subjects for investigation. We know nothing of the ψυχή, if there be a ψυχή, and can only wait and hope with regard to it. In the words of Pasteur, "Les deux domaines sont distincts, et malheur à celui qui veut les faire empiéter l'un sur l'autre dans l'état si imparfait des connaissances humaines." Those who would thus "faire empiéter," call themselves "medico-psychologists," and regard their environment as profound when it is only muddy. We may leave it to Dante to dismiss them from consideration.

"Non ragioniam di lor, ma guarda e passa."

